# A Few More “Therapeutic Hints”

**Published:** 1895-08

**Authors:** C. M. Boger

**Affiliations:** Parkersburg, W. Va.


### A FEW MORE "THERAPEUTIC HINTS."

C. M. Boger, M. D., Parkersburg, W. Va.

Sensation of heaviness in glands, especially in mammæ, Iodum. This is a key-note. Vertigo due to scanty or suppressed urine, Anacardium. Chill starting in bladder or neck of bladder, *Sarsaparilla.* Chill starts in small of back going both up and downwards, Ruta-grav. Rheumatism of the hip joints, especially when throbbing (Stram.) is present, *Mag-m.*

